# More User-Friendly Metrics

**DOI:** 10.21470/1678-9741-2019-0211

**Published:** 2019

**Authors:** Erwin Krauskopf

**Affiliations:** 1 Universidad Andres Bello, Facultad de Ciencias de La Vida, Santiago, Chile.

Dear editor,

I read with great interest the latest editorial published by the Brazilian Journal of Cardiovascular Surgery (BJCVS)^[1]^ in which the authors presented their views about the impact factor provided by the Journal Citation Report (JCR). However, I believe that the readers of this journal should be aware that BJCVS is also indexed by another global citation database, Scopus. As JCR generates a list of journals indexed by Web of Science, SCImago generates annually a list of journals indexed by Scopus, which are sorted by their SCImago journal rank (SJR) for each of its subject categories. Hence, according to data from SCImago, BJCVS is categorized as Q3 in three subject categories: “Cardiology and Cardiovascular Medicine”, “Medicine (miscellaneous)”, and “Surgery”.

As the journal has been indexed by Web of Science since 2016, I extracted data published by BJCVS from Scopus for the same time period to establish other journal’s attributes.

First, I searched for the documents published by other journals that cite articles published by BJCVS. I sorted the citing journals by the total number of BJCVS articles cited and determined to which quartile each of the journals belonged, according to their SJR2017. As Table 1 shows, half of the citing journals belong to the first quartile, fact that corroborates the quality of the research being published by BJCVS.

**Table 1:** Ranking of the top-10 journals citing articles published by BJCVS, based on the total number of cited articles. Quartile distribution was estimated using the SJR2017.

Source Title	Number of documents	Quartile
Brazilian Journal of Cardiovascular Surgery	161	Q3
Journal of Cardiothoracic and Vascular Anesthesia	35	Q2
International Journal of Cardiology	22	Q1
Plos One	21	Q1
Journal of Thoracic and Cardiovascular Surgery	20	Q1
Annals of Thoracic Surgery	19	Q1
Arquivos Brasileiros de Cardiologia	19	Q3
Interactive Cardiovascular and Thoracic Surgery	14	Q2
Journal of Cardiothoracic Surgery	13	Q2
Journal of Vascular Surgery	13	Q1

BJCVS=Brazilian Journal of Cardiovascular Surgery; SJR=SCImago journal rank

As the editorial mentions, most of the contributions accepted by the journal are written by Brazilian researchers from Brazil, followed at a distance by researchers from Turkey, China, United States of America, and India. However, a closer look at the country of affiliation of those researchers that use the information published by BJCVS reveals that 480 articles were written by Brazilian researchers, 341 were authored by researchers from United States of America, 191 were written by Chinese researchers, 89 were authored by British researchers, and 80 were written by German researchers. This information is relevant to readers as it confirms the international visibility of the work being published by BJCVS.

Currently, many members of the research community are considering the use of new metrics to evaluate journals where to submit their manuscripts, following the guidelines set at the San Francisco Declaration on Research Assessment, also known as DORA^[2]^. Perhaps it is time to begin using metrics that are more meaningful to the researchers.
